# Muc5b Is the Major Polymeric Mucin in Mucus from Thoroughbred Horses With and Without Airway Mucus Accumulation

**DOI:** 10.1371/journal.pone.0019678

**Published:** 2011-05-13

**Authors:** Karine Rousseau, Jacqueline M. Cardwell, Emma Humphrey, Richard Newton, David Knight, Peter Clegg, David J. Thornton

**Affiliations:** 1 Wellcome Trust Centre for Cell-Matrix Research, Faculty of Life Sciences, University of Manchester, Manchester, United Kingdom; 2 Animal Health Trust, Lanwades Park, Kentford, Newmarket, United Kingdom; 3 Department of Musculoskeletal Biology, University of Liverpool, Neston, United Kingdom; University of California Merced, United States of America

## Abstract

Mucus accumulation is a feature of inflammatory airway disease in the horse and has been associated with reduced performance in racehorses. In this study, we have analysed the two major airways gel-forming mucins Muc5b and Muc5ac in respect of their site of synthesis, their biochemical properties, and their amounts in mucus from healthy horses and from horses with signs of airway mucus accumulation. Polyclonal antisera directed against equine Muc5b and Muc5ac were raised and characterised. Immunohistochemical staining of normal equine trachea showed that Muc5ac and Muc5b are produced by cells in the submucosal glands, as well as surface epithelial goblet cells. Western blotting after agarose gel electrophoresis of airway mucus from healthy horses, and horses with mucus accumulation, was used to determine the amounts of these two mucins in tracheal wash samples. The results showed that in healthy horses Muc5b was the predominant mucin with small amounts of Muc5ac. The amounts of Muc5b and Muc5ac were both dramatically increased in samples collected from horses with high mucus scores as determined visually at the time of endoscopy and that this increase also correlated with increase number of bacteria present in the sample. The change in amount of Muc5b and Muc5ac indicates that Muc5b remains the most abundant mucin in mucus. In summary, we have developed mucin specific polyclonal antibodies, which have allowed us to show that there is a significant increase in Muc5b and Muc5ac in mucus accumulated in equine airways and these increases correlated with the numbers of bacteria.

## Introduction

The gel-forming, epithelial mucins are large polymeric glycoproteins that are a major structural component of the mucus barrier, which forms a protective interface against the external environment. Their major role is to maintain hydration of the airway epithelium and to provide a milieu to entrap external agents, both biological (allergens and bacteria) and chemical (particles and pollutant gases), which can then be removed from the airways by mucociliary clearance. The two most common inflammatory respiratory syndromes of horses are recurrent airway obstruction (RAO) and inflammatory airway disease (IAD). These conditions are characterized by airway mucus over-production and impairment in mucociliary clearance. RAO, associated with chronic exposure to environmental allergens, predominantly affects middle-aged to older (usually ≥7 years old) housed horses [1] with the prevalence of the condition increasing with age [1], [2], [3], [4]. Reduced airflow is associated with bronchoconstriction, mucus hypersecretion and airway neutrophilia [2], [5], [6], [7], [8]. Clinical signs range from slight exercise intolerance to dyspnoea at rest. Episodes may be reversed or alleviated by drug therapy or changes to management resulting in improved air quality [5], [9], [10]. Young racehorses in training suffer from IAD, a condition that also involves airway neutrophilia and increased amounts of tracheal mucus [11], [12], [13], [14]. Clinical signs of IAD include coughing [11], [12] and poor racing performance [15], [16], [17], [18], [19].

We have shown previously that Muc5b and Muc5ac are the predominant mucins in airway secretion from healthy horses [20]. However, while mucus accumulation in the airways is associated with IAD and RAO and contributes to the pathological symptoms, little is currently known about the gel-forming mucins in these conditions and how they contribute to the aberrant clearance of mucus. As in the horse, the orthologous mucins, MUC5B and MUC5AC, are the major gel-forming mucins in human airway mucus [21], [22]. In normal airway epithelium the expression of the two mucins is cell specific; MUC5B is mainly expressed by the submucosal glands, and MUC5AC expressed by the goblet cells at the surface epithelium [22], [23]. In human pathological conditions, such as asthma, chronic obstructive pulmonary disease (COPD) and cystic fibrosis (CF), mucin expression is altered with an increase in the amounts of both MUC5B and MUC5AC. Furthermore, MUC5B was more abundant in mucus obstructing the airways [24], [25], [26]. In addition, *in vitro* studies have also shown that the regulation of these two mucins can be altered by inflammatory mediators such as cytokines, and directly by external challenges such as bacteria [27], [28], [29], [30], [31].

The studies on human airway mucus have highlighted that the gel-forming mucin composition and concentration in mucus likely impacts on its efficient clearance from the respiratory tract. While in-roads have been made in the understanding of human airway mucus there are many unanswered questions relating to equine airway mucins and mucus. For example, what are the sites of synthesis of these mucins? Which mucins are up-regulated in hypersecretory conditions, in particular in IAD? What is the composition of mucus that accumulates in the airways? To address these issues we have raised and characterised antisera specific to equine Muc5b and Muc5ac. We have shown that in normal equine trachea, Muc5ac and Muc5b are products of both epithelial goblet cells and cells in the submucosal glands. Both mucins contributed to mucus accumulated in the airways of a cohort of Thoroughbred racehorses, although Muc5b was generally present in higher amounts.

## Results

In our previous work, we used mass spectrometry to demonstrate that Muc5b and Muc5ac are the major polymeric mucins in equine airway mucus [20]. However, no tools were available to distinguish between these two mucins in mucus, or in respiratory tissue. In the present study, our aims were to develop antibody probes specific for Muc5b and Muc5ac in order to determine the sites of synthesis of Muc5ac and Muc5b in equine upper airways, to characterise the biochemical properties of the two mucins, and to determine the relative amounts of Muc5ac and Muc5b in mucus collected from horses with and without signs of mucus hypersecretion.

### Characterisation of mucin-specific antisera

Polyclonal antisera were raised against peptides located within the putative non-glycosylated, cysteine-rich domains of the central regions of Muc5b (MANeq5b-I; DEDYPTSEKAGGDIEC) and Muc5ac (MANeq5ac-I; GIDSGDFDTLENLR). These peptides were selected after analysis of the putative equine Muc5ac and Muc5b gene sequences [20] to ensure that each sequence was specific for its respective mucin. To test if the antisera could discriminate between Muc5ac and Muc5b we performed immunohistochemical analysis of equine tissues that differentially express the two mucins. Salivary and gastric tissues were studied since Real-Time (RT) PCR data showed that *Muc5ac* was highly expressed in gastric tissue and *Muc5b* was not detected, whereas *Muc5b* was expressed in salivary glands and *Muc5ac* was not detected (Table 1). This is in accordance with published data from horse, human and other species [20], [32], [33].

**Table 1:** Real-Time PCR results obtained from mRNA extracted from salivary glands, trachea and stomach showing differential expression of the *Muc5b* and *Muc5ac* in these tissues.

	*Muc5ac*	*Muc5b*
Stomach	19.06 (±4.8)	ND
Trachea	0.108 (±0.13)	3.541 (±1.8)
Salivary gland	ND	18.3 (±3.2)
ND: not detectable		

The data shown are raw expression normalized to GAPDH and SDHA housekeeping genes.

**Figure 1:**
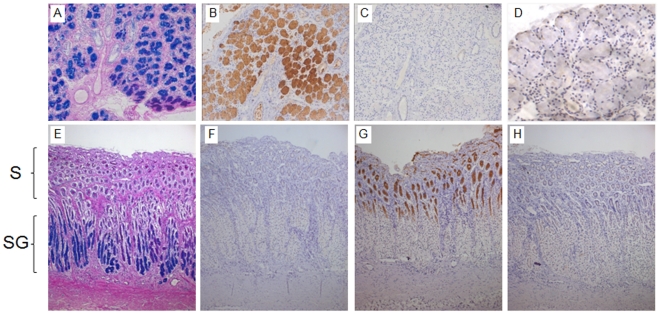
Histological and immunohistochemical staining of equine salivary gland and stomach tissues. Panels A (salivary glands) & E (stomach) show glycoprotein staining with PAS-AB. Sections from salivary glands (B–D) and stomach (F–H) were used to characterise the purified antisera. Panels B & F show the staining of equine salivary gland tissue and stomach with MANeq5b-I and panels C & G show staining of these tissues with MANeq5ac-I. Panels D & H show inhibition of antisera reactivity with the peptides used for immunisation. For the stomach sections, S indicates the location of the surface mucous cells, and SG indicates the location of the submucosal gland tissue. All pictures were taken at 20X magnification apart from panel D which was taken at 40X.

Sections of dorsal buccal salivary glands and gastric tissue were examined. PAS-AB staining of these tissues was first used to identify the glycoprotein containing cells. In dorsal buccal salivary glands, there was strong staining of the majority of the cells with PAS-AB (Figure 1A) and in the glandular gastric tissue, surface mucous cells and cells in the deeper glands showed staining with PAS-AB (Figure 1E). Similar sections were stained with protein-A purified MANeq5b-I and MANeq5ac-I sera. As expected from the mRNA data, salivary gland cells were strongly stained with MANeq5b-I (Figure 1B) but not stained with MANeq5ac-I (Figure 1C), whereas gastric tissue showed no staining with MANeq5b-I (Figure 1F) but showed strong staining of the surface epithelial cells with MANeq5ac-I (Figure 1G). Pre-incubation of each antiserum with the peptide used for immunisation abolished immunohistochemical staining (Figure 1D and H), supporting their ability to discriminate between the two mucins.

### Biochemical characterisation of Muc5ac and Muc5b

**Figure 2:**
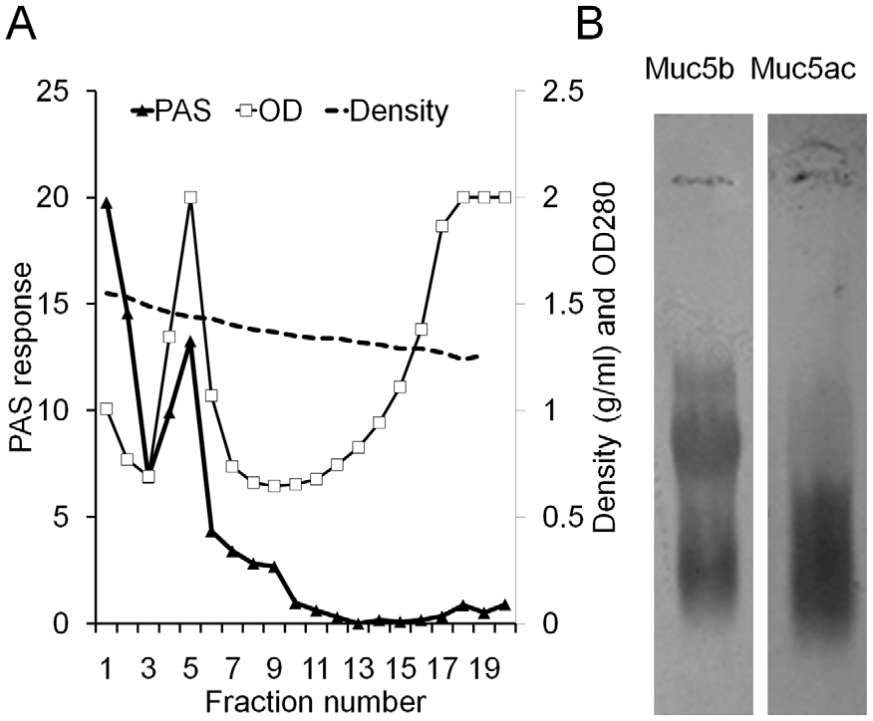
Purification of Muc5ac and Muc5b by caesium chloride density gradient centrifugation. Tracheal washes were dissolved in equal volume of 8 M guanidinium chloride and the mucins separated from other proteins by CsCl density gradient centrifugation. Panel A: Fractions were analysed for optical density at 280 nm (open squares) and the mucin containing fractions were determined by PAS staining (black triangles). The density of each fraction was determined by weighing (dashed line). Panel B: MANeq5b-I and MANeq5ac-I antisera were used to probe a Western blot after agarose gel electrophoresis of the mucins in fraction 5.

In order to characterise Muc5ac and Muc5b, four tracheal wash samples from horses showing signs of mucus hypersecretion were pooled, solubilised in 4M guanidinium chloride and analysed by caesium chloride density gradient centrifugation (buoyant density), anion exchange chromatography (charge distribution) and rate-zonal sedimentation (size distribution). PAS staining of fractions across the density distribution (Fig. 2A) showed that mucins were enriched in the high buoyant density fractions (1.37–1.55 g/ml). The mucins in fraction 5 were reduced and carboxymethylated and subjected to agarose gel electrophoresis (Fig. 2B). A Western blot of the gel probed with MANeq5b-I showed three bands. In contrast, MANeq5ac-I detected a single, broad band that migrated in a similar position to the fastest migrating Muc5b-band.

**Figure 3:**
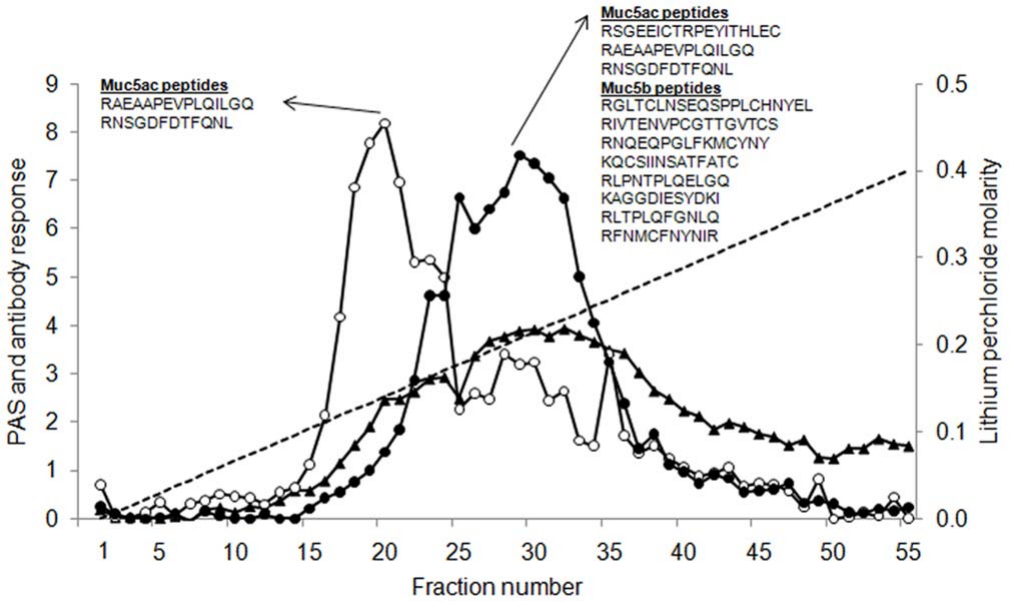
Separation of Muc5b and Muc5ac by anion exchange chromatography. Purified respiratory mucins were reduced and carboxymethylated and dialysed into 6 M urea prior to anion exchange chromatography on a Resource Q column. Aliquots of each fraction were analysed by PAS-staining (black triangles) and for reactivity with MANeq5b-I (black circles) and MANeq5ac-I (open circles). The dotted line indicates the linear gradient of lithium perchlorate. Inset shows Muc5ac and Muc5b peptides identified by tandem mass spectrometry analysis of fractions 20 and 29.

To investigate whether the separation between Muc5ac and Muc5b was due to the charge density of the molecules the reduced and carboxymethylated mucin preparation was subjected to anion exchange chromatography (Fig. 3). This method has been used previously to separate human airway mucins [34], [35]. The total glycoprotein profile was assessed by PAS staining and showed that the molecules had a broad distribution of charge density. Fractions from across the chromatogram were also analysed by immunoblotting using MANeq5ac-I and MANeq5b-I (Figure 3). The reactivity profiles of the two antisera showed different but overlapping distributions. MANeq5ac-I-reactive material was present in two peaks; one major peak (fractions 15-25) and a more minor peak (fractions 26–40). In contrast, MANeq5b-I reactive material was confined to a single, broad peak (fractions 16–40). As expected from the antibody profiles, tandem mass spectrometry analysis of tryptic peptides derived from the Muc5ac-enriched peak (fraction 20) and the Muc5b-enriched peak (fraction 29) showed that Muc5ac was present in fraction 20 (2 Muc5ac peptides, no Muc5b peptides) and Muc5b and Muc5ac were present in fraction 29 (8 Muc5b peptides, 3 Muc5ac peptides). These data indicate that the two antibody probes can be used to discriminate between Muc5ac and Muc5b in immunoblotting experiments. Furthermore, the major population of Muc5ac is more lowly charged than Muc5b, with Muc5ac showing evidence of different charged variants (or glycoforms).

**Figure 4:**
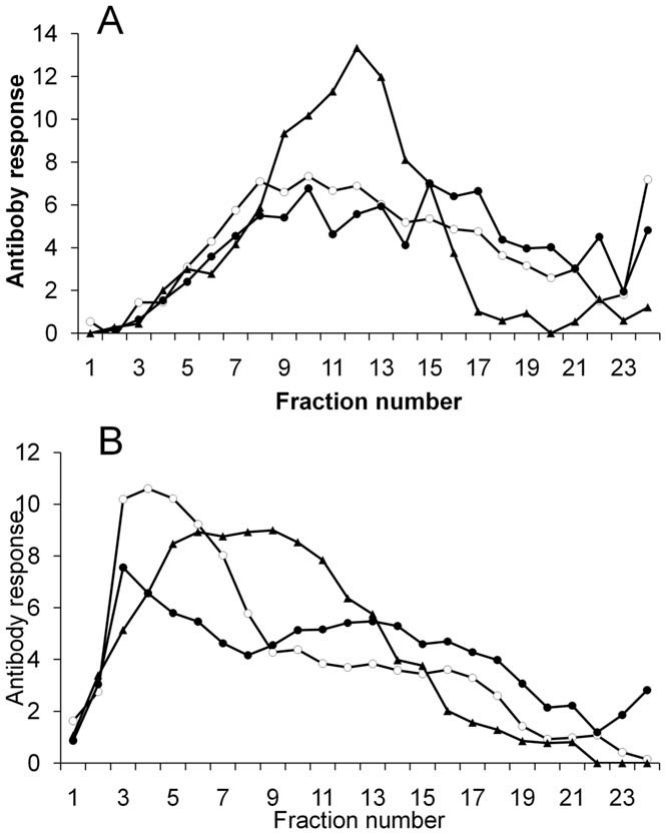
Sedimentation analysis of Muc5ac and Muc5b. Unreduced (panel A) and reduced mucins (panel B) were subjected to rate-zonal centrifugation on 6-8 M guanidinium chloride gradients. Fractions were analysed for glycoprotein content by PAS-staining (black triangles), and for reactivity with MANeq5b-I (black circles) and MANeq5ac-I (open circles). Fractions 1 and 24 represent the top and bottom of the gradient, respectively.

To analyse the size distributions of Muc5ac and Muc5b mucins, airway mucus (pooled from 4 horses with airway mucus accumulation) was subjected to rate-zonal centrifugation, before and after treatment with a reducing agent (10 mM DTT). The unreduced Muc5ac and Muc5b mucins were characterized by a broad range of sedimentation rates, characteristic of a polydisperse distribution of mucins (Figure 4A). After reduction, the mucins exhibited slower sedimentation rates. Interestingly, there was a separation of the PAS-reactivity from a slower sedimenting population of molecules that was highly reactive with the mucin probes (Figure 4B).

### Determination of the site of mucin synthesis

**Figure 5:**
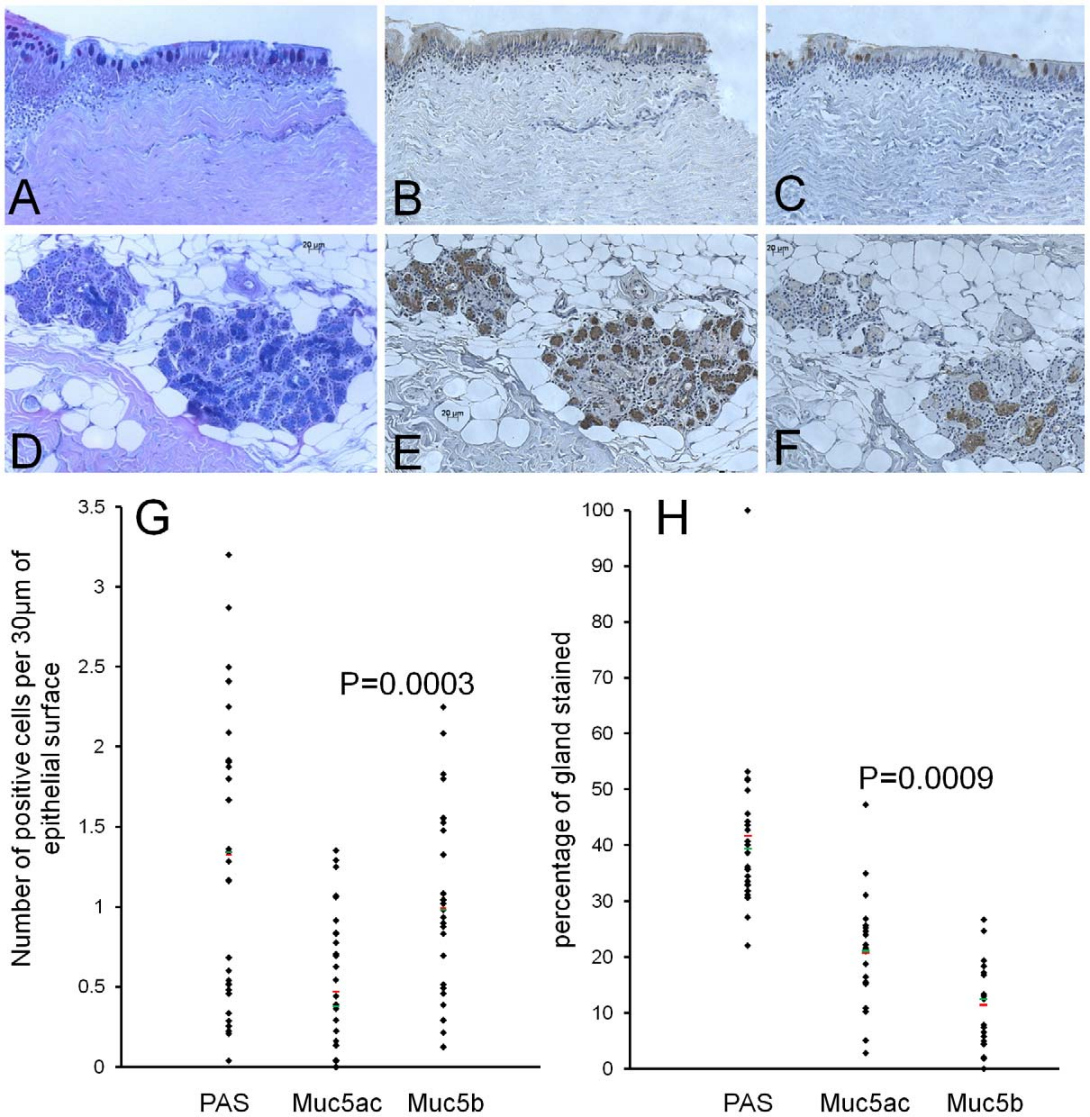
Histological and immunohistochemical staining of normal equine tracheal tissue. 5 µm sections of tracheal tissue were stained with (panels A and D) PAS, (panels B and E) MANeq5ac-I and (panels C and F) MANeq5b-I. In panels B, C, E and F the sections were reduced and carboxymethylated prior to staining with protein-A purified antibodies. Panels A, B and C show the staining of the surface epithelium while panels D, E and F show staining of the submucosal glands. Panels G and H show the quantitation of staining for PAS and the two antibodies. The analysis of the staining was performed on three different horses. For the surface epithelium the goblet cell staining was determined for 9×750 µm lengths of epithelium. For submucosal gland staining a minimum of 6 glands per horse were analysed. The red line represents the average and the green line the median.

Immunohistochemical analysis of gastric and salivary mucins, as well as the biochemical analysis of the mucins, has demonstrated the specificity of the two polyclonal antisera. Therefore, we used the two mucin-probes to analyse the sites of synthesis of mucin production. Staining of normal tracheal tissue with PAS-AB (Figure 5A) showed individual epithelial goblet cells stained both pink (neutral mucins) and/or blue (acidic mucins). Within the glands the PAS-AB staining is predominantly blue, indicating that the mucins are mainly acidic (Figure 5D). Serial sections stained with protein-A purified MANeq5ac-I (Figure 5B and 5E) and MANeq5b-I (Figure 5C and 5F) showed that both mucins are expressed in epithelial goblet cells (Figure 5B and 5C) and submucosal gland cells (Figure 5E and 5F). Analysis of the staining showed that the surface epithelium contained more goblet cells positive for Muc5b than Muc5ac (approx. 1 cell staining for Muc5b and approx. 0.5 cell staining for Muc5ac per 30 µm of surface epithelium, p = 0.0003, Figure 5G). Furthermore, a higher percentage area of the glands stained with Muc5ac than Muc5b (21% and 11.5% respectively, p = 0.0009, Figure 5H). It is not clear whether both mucins are present in the same cells. For both mucins, the antibody staining was abolished by inhibition with the peptide used for immunisation.

### Muc5b and Muc5ac in tracheal wash samples from thoroughbred horses in training

**Figure 6:**
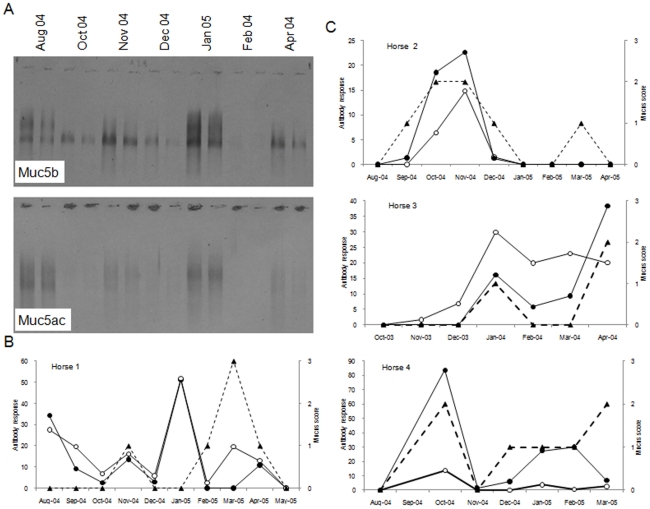
Variation of Muc5b and Muc5ac composition of tracheal washes from Thoroughbred horses in training. (Panel A): Seven different tracheal wash samples collected from a single horse (Horse 1) in August, October, November, December 2004 and January, February and April 2005 were reduced and carboxymethylated and subjected to agarose gel electrophoresis. Two dilutions of each sample (1/5 and 1/10) were loaded onto a 0.7% agarose gel. A Western blot of the gel was probed for Muc5b with MANeq5b-I and Muc5ac with MANeq5ac-I. (Panels B and C): Graphical representation of the mucus score attributed to each sample by endoscopy at the time of collection (black triangle and dotted line) and MANeq5b-I (black circles) and MANeq5ac-I reactivity (open circle) after Western blotting across the sampling period for horse 1 (panel B) and for three other horses (panel C).

The mucin-specific antisera were employed in a Western blotting assay to analyse the Muc5ac and Muc5b content of respiratory mucus. We first investigated the mucin composition in tracheal washes from four horses over the course of several months. The Western blot results for seven tracheal wash samples collected from one of the horses is shown in Figure 6A. The blot showed a wide variation in the amount of the two mucins across the sampling period and, furthermore, there was a variation in the number and electrophoretic mobility of mucin bands. In general, the variation in the amount of the mucins followed the amount of mucus in each sample, determined endoscopically at the time of sample collection (Figure 6B & C). However, this is not always the case and for some tracheal wash samples the mucin content did not follow the amount of mucus.

**Figure 7:**
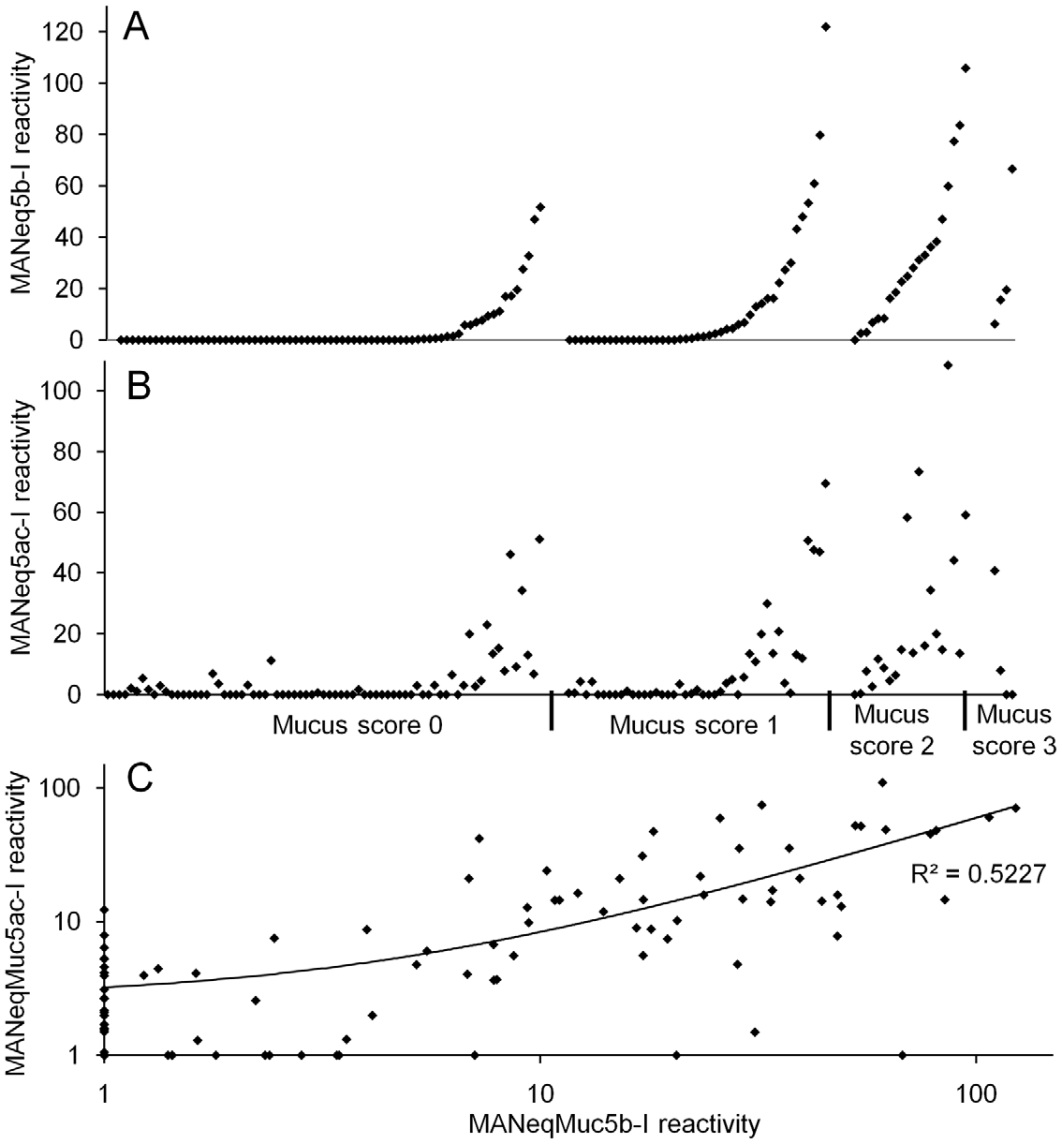
Correlation between mucus score and mucin amounts of tracheal washes from Thoroughbred horses in training. Graphical representation of the mucus score versus the MANeq5b-I (panel A) and MANeq5ac-I (panel B) reactivity for each tracheal wash sample. On graphs A and B, the samples are ordered first according to their mucus score then according to their MANeq5b-I reactivity. ANOVA analysis showed that there was a strong association between the increase of the mucus score and the increase of the mucin antibody reactivity (p<0.001 for both mucins). For the ANOVA analysis, samples from mucus score 2 and 3 were grouped due to low sample number in the mucus score 3 group. (Panel C): Graphical representation of the correlation between Muc5b and Muc5ac content of tracheal wash samples (p<0.001).

We next analysed the Muc5ac and Muc5b composition of 144 tracheal wash samples, collected from thoroughbred racehorses as part of a wider study of IAD [36], [37]. In the first series of experiments, a 20 µl and a 40 µl aliquot of each sample was analysed using the Western blot procedure. Approximately 50% of the samples showed no staining with the antisera. These were below the level of detection of the assay and were not studied further (62 samples for Muc5ac and 71 samples for Muc5b). The samples that gave a positive response in the assay were further analysed by Western blotting (each sample was diluted 1∶5, 1∶10, 1∶20 and 1∶40) and the average staining intensity for MANeq5b-I and MANeq5ac-I for each sample was determined. The analyses resulted in a value of antibody reactivity for each samples reflecting Muc5ac and Muc5b content. The complete set of data is shown in supplementary data (Table S1). The mucin content showed a strong, positive association with the mucus score, although this was not the case for all samples. Samples with a high mucus score (mucus score 2 and 3) were found to have the highest amounts of Muc5b and Muc5ac (p<0.001 for both) (Figure 7A & B). There was also a strong, positive correlation between Muc5b and Muc5ac (p<0.001) (Figure 7C), showing that samples containing a high amount of Muc5b also had higher amounts of Muc5ac (Figure 7C).

**Figure 8:**
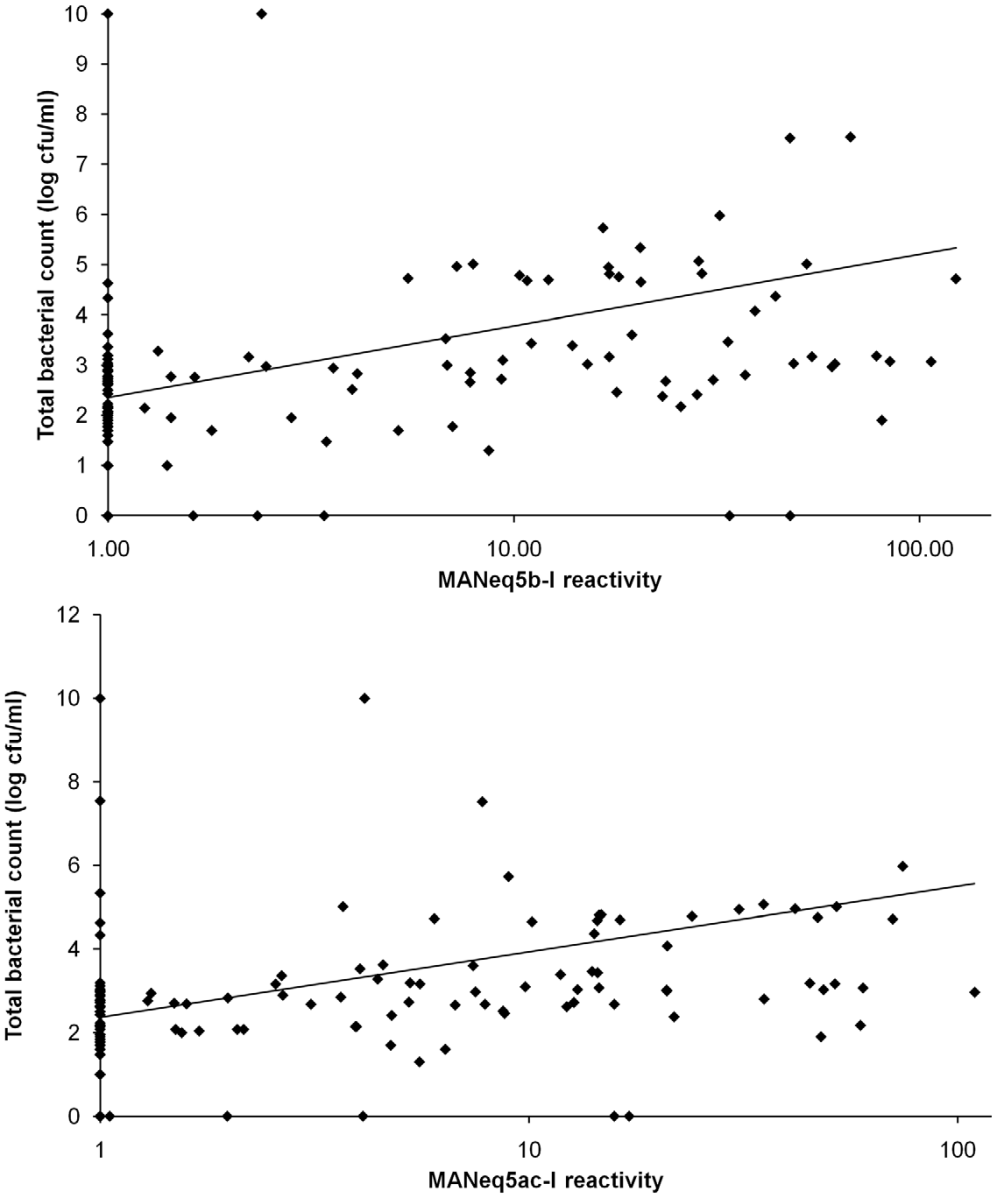
Correlation between mucin specific antibody reactivity and bacterial count. Top panel: Graphical representation of the association between the MANeq5b-I response and the total bacterial counts (p<0.001) Bottom panel: graphical representation of the association between the MANeq5ac-I response and the total bacterial counts (p<0.001).

The total number of bacteria present in each tracheal sample was also analysed against its mucin content showing that the samples with increased amounts Muc5b and Muc5ac also had higher total bacterial counts (Figure 8, p<0.001)). However, there was no specific association with any individual bacterial species such as *Streptococcus zooepidemicus*, *S. viridians*, non-haemolytic Streptococcus spp or Staphylococcus spp (data not shown).

### Muc5b is the main mucin in healthy and diseased samples

While the compositional analyses reflect the mucin content of each sample, they are not a quantitative measure of the amount of mucin. In particular, the analyses are dependent on the sensitivity of each antiserum. In order to establish the range of detection of MANeq5ac-I and MANeq5b-I mucin, solutions of known concentration were prepared. Horse gastric mucus was used as the source of Muc5ac and horse saliva as the source of Muc5b. Mucins were purified by caesium chloride density gradient centrifugation and their mucin content, measured by refractometry, was 73.5 µg/ml (gastric mucins) and 80 µg/ml (salivary mucins). Tandem mass spectrometry showed that the gastric mucin preparation contained Muc5ac. Surprisingly, although Muc6 is expressed in the stomach along with Muc5ac, no peptides for Muc6 were identified by tandem mass spectrometry, indicating that Muc6 is not a major component of our preparation. The salivary preparation contained both Muc5b and Muc19 mucins. The peak intensities of the three most abundant peptides from Muc5b and Muc19 were used to determine the ratio of the two in the purified mucin preparation in a similar manner to that used in Water's Expression^E^ approach [38]. Since there are no reports of the molecular mass for equine Muc19 or Muc5b the following three assumptions were made to estimate the relative amounts of the two mucins in the preparation. Firstly, the estimated molecular weight of the Muc19 polypeptide was approximately 840 kDa based on the recent report by Chen and colleagues of the estimated full-length sequence of the human *MUC19* gene [39]. Secondly, the estimated molecular weight of the Muc5b polypeptide was approximately 570 kDa based on the database sequence of the human MUC5B polypeptide, Q9HC84. Thirdly, both mucins had a similar percentage of their mass in the form of carbohydrate. Based on these assumptions in conjunction with the mass spectrometry data we calculated the mass ratio of Muc5b/Muc19 ratio to be 1.3 (±0.2), which indicates that the salivary mucin solution contains approximately 45 µg/ml of equine Muc5b.

**Figure 9:**
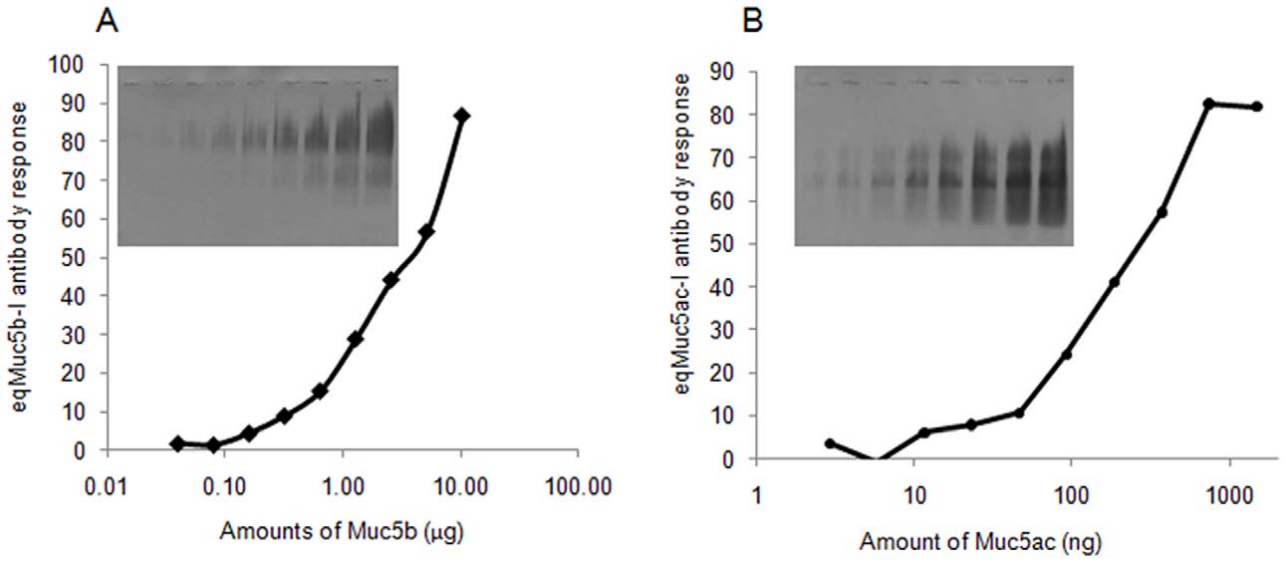
Determination of the sensitivity of the MANeq5b-I and MANeq5ac-I antisera. Reduced and carboxymethylated mucin standard solutions, prepared from saliva (Muc5b 10-0.02 ng) and from equine stomach mucus (Muc5ac, 1480-2.9 ng), were electrophoresed on 0.7% agarose gels. Western blots of the gels were probed with MANeq5b-I (inset in panel A) and MANeq5ac-I (inset in panel B), the intensity of the staining was measured by reflectance densitometry and is plotted against the amount of mucin.

A range of amounts of the two-mucin standard solutions was electrophoresed and Western blots of the gels were probed with either MANeq5ac-I or MANeq5b-I. The response curves generated show that eqMuc5ac-I recognises substantially lower levels of mucin than the eqMuc5b-I (Figure 9). MANeq5b-I antibody detected between 0.1 to 10 µg of Muc5b while MANeq5b-I detected between 10 to 1000 ng of Muc5ac (Figure 9). A retrospective analysis of the data generated above showed that Muc5b is the major mucin in the airway mucus, and that although the amounts of both mucins could be increased by more than 10-fold in tracheal wash samples from horses with mucus accumulation, Muc5b remained the most abundant mucin in the vast majority of samples analysed.

## Discussion

We have generated antisera specific to the two major equine airways polymeric mucins, Muc5ac and Muc5b. These probes have been employed to distinguish between Muc5ac and Muc5b by using immunohistochemistry of tissue sections, and by immunoblotting after fractionation of tracheal mucus. Immunohistochemical staining has shown that in normal trachea Muc5b and Muc5ac are both produced by cells in the submucosal glands and surface epithelial goblet cells. At present, the relative contribution of the submucosal glands and the goblet cell secretions to the mucus layer is not known. The analysis of the antibody staining data showed that more surface epithelial goblet cells stained for Muc5b than Muc5ac. In contrast, the submucosal glands showed more staining for Muc5ac than Muc5b. Of course, due to the difference in sensitivity of our antisera (discussed below) low amounts of Muc5b in the glands might not be efficiently detected. The distribution of Muc5b and Muc5ac in the horse is unexpected, since this localisation data contrasts with the distribution of the orthologues of these two mucins in human upper airways, where MUC5B is expressed mainly in mucous cells in the submucosal glands and MUC5AC is produced mainly by the surface goblet cells [22], [40]. However, we have only examined normal tracheal tissue and have no data regarding the cellular origins of these two mucins in the lower airways, nor in diseased tissues. It will be important to determine the site of synthesis of Muc5ac and Muc5b in horses with airway obstruction to inform future therapeutic developments targeted at modulating mucin production in horses with respiratory disease.

Analysis of Muc5ac and Muc5b by rate-zonal centrifugation showed that both mucins had heterogeneous sedimentation profiles, indicative of polydisperse size distributions. This is consistent with previous findings which showed that equine respiratory mucins are polymeric and heterogeneous in size [20] as are their human counterparts [26], [34], [35], [41]. After cleavage of disulphide bonds, Muc5ac and Muc5b showed a marked decrease in sedimentation rate, consistent with the effect of reducing agents on human MUC5AC and MUC5B [26], [34], [35], [41]. It is noteworthy that after reduction, two populations of Muc5ac and Muc5b are observed. The population with higher sedimentation rate most likely represents the reduced mucin monomers (subunits; [26], [34], [35], [41]), whereas, the more slowly sedimenting molecules may represent differently glycosylated populations of Muc5ac and Muc5b, or protein-rich fragments of the mucin polypeptides generated by proteolytic modification, as demonstrated for human polymeric mucins [26], [34], [35], [41].

Agarose gel electrophoresis identified at least three populations of Muc5b, but these were not apparent in all samples. Under the same conditions, human MUC5B showed two forms, which resulted from differences in their charge density (low and high charge glycoforms; [25], [26]). However, anion exchange chromatography of the equine mucins identified a single, broad peak for Muc5b with no obvious evidence for distinct populations. Interestingly, one heterogeneous band, with variable mobility between horses, was observed after electrophoresis of Muc5ac. In contrast, anion exchange chromatography identified two peaks. The major peak exhibited a lower charge density than the more minor population, and the majority of Muc5ac had a low charge density but a high electrophoretic mobility. Therefore, unlike human MUC5AC and MUC5B, the electrophoretic mobilities of the equine orthologues do not correlate with charge density (as assessed by anion exchange chromatography). These findings show that the electrophoretic behaviour of the equine mucins is not simply explained by their charge density. Other factors are clearly important. For example, the molecular weight of the mucins may impact on their electrophoretic migration. This could be affected by the extent of glycosylation of the mucins or their incomplete reduction that may have yielded multimeric species rather than mucin monomers. Also, the binding of SDS to the polypeptides may be different. This could be due to inherent differences in the primary sequences of the mucin poypeptides or might arise by proteolytic cleavage. These analyses have shown that the major population of Muc5ac has a lower average charge density than Muc5b. However, it is not known how this relates to biological function. Potential differences in the glycosylation of the mucins are likely to result in different interactions with respiratory pathogens or with other components of the mucus. Whether the latter impacts on the protective nature of mucus in terms of mucociliary clearance remains to be elucidated. In humans, changes in the glycosylation of MUC5B have been shown to occur in CF and COPD, and a specific glycoform of MUC5B (low charge glycoform) has been found to be more prevalent [25], [26]. Again, it is not known how this impacts on mucus function. However, a low charge MUC5B glycoform was the predominant mucin in mucus that obstructed the airways in an individual who died in *status asthmaticus* [42].

Analysis of the mucin composition in serial tracheal washes over the course of 12 months revealed marked variations in the content of Muc5ac and Muc5b. This trend generally reflected the amount of mucus in the airways measured visually at the time of tracheal washing. However, at some time-points the two did not match and a small amount of mucus was not associated with a low level of the mucins, and vice versa. This could be explained in a number of ways; (i) other unidentified macromolecules present in the secretion (eg. hyaluronan or proteoglycans that have been observed in mucus produced in human airway disease [43], [44], [45] may result in the visual appearance of mucus, (ii) the washing procedure did not remove the mucus from the airways (adherent mucus has been observed in the airways of animal models of cystic fibrosis [46], [47]), (iii) the mucus was in a form not readily identified by endoscopy. This warrants further investigation in the future. It is also noteworthy that amongst the samples from horses with no visible mucus in their trachea, some were found to have detectable amounts of mucins (Figures 6 & 7). This highlights a discrepancy between the amount of mucus identified visually by endoscopy and the amount of mucins determined by immunoblotting and might reflect a difference in the mucus properties, or at least in mucus appearance. However, it could be that the tracheal wash technique samples areas of the airway that are not accessible with an endoscope, indicatingthat the mucin specific analyses from tracheal washes could be of value for the diagnosis of lower airway infection.

Analysis of the Muc5ac and Muc5b mucin composition of mucus accumulated in the airways of a cohort of thoroughbred racehorses showed that the Muc5b and Muc5ac content increased with increasing amounts of mucus. Furthermore, the amount of both mucins was increased in samples showing an increase in the number of bacteria in the mucus. These observations may not be too surprising and while the amounts of both mucins are increased, the western blotting analyses were at best semi-quantitative and do not reveal the actual amount of the mucins and their fold-increase. Therefore, we addressed this issue retrospectively by preparing standard solutions of Muc5ac and Muc5b to characterise further the polyclonal antisera. This showed that MANeq5ac-I was able to detect approximately 20-fold less mucin than MANeq5b-I and thus Muc5b was the major mucin in all the samples analysed. We are aware that this conclusion relies on several assumptions that were made in determining the concentration of the mucin standards. In the case of the gastric mucin preparation (Muc5ac standard) only Muc5ac was identified by mass spectrometry. This was unexpected because in the human MUC6 is highly expressed in the deeper glands of the stomach and we therefore expected to find Muc6 in our preparation [48]. However, in agreement with our finding MUC5AC but no MUC6 was detected in mucus harvested by gentle scraping of the human gastric epithelium [49], a similar approach to that used here to collect the equine mucus. Of course, we cannot conclude that Muc6 was absent from the gastric mucin preparation but we can conclude that Muc5ac was the major mucin in the standard solution. Consequently, we may have slightly overestimated the amount of Muc5ac in the standard solution, resulting in an underestimation of the amount of Muc5ac in the samples. Unlike the Muc5ac standard, the Muc5b standard solution (prepared from saliva) is a mixture of the two mucins Muc5b and Muc19. Ideally we would have separated the two mucins but this was not possible using current separation methodologies (data not shown). Therefore, we calculated the concentration of each mucin in the standard solution from tandem mass spectrometry data and employing the current information on human MUC5B and MUC19 mucins to predict the molecular weight of the mucin polypeptides [39], [50], [51].

Despite the issues mentioned above, the data are in accordance with the real time PCR data presented here, and with the previously published mass spectrometry analyses of equine airway mucins indicate that Muc5b is the major polymeric mucin expressed [20]. This is also in agreement with observations made in other animals. For example, in unchallenged mice airways it has been reported that Muc5b is more abundant than Muc5ac [52], [53] and Young and co-workers have reported that *Muc5b* mRNA was 40 fold more abundant than *Muc5ac* mRNA [54].

While this is the first study to report on the gel-forming mucin composition of equine airway mucus, one should be aware that there are limitations to the approach employed. First of all, it is worth pointing out that for a number of samples no mucin was detectable. However this is dependent on the sensitivity of the antibody used to detect the mucins. In the case of Muc5b, we found that the antibody is not very sensitive and cannot detect reliably amounts lower than 100 ng, while the eqMuc5ac antibody is more sensitive and can detect as little as 10 ng. This means that in the samples where mucus can be seen, but no mucins were detected, it is possible that the mucus present is composed mainly of Muc5b, in amounts lower than the sensitivity of our assay. Another potential problem is cross-reactivity of the antisera with other polymeric mucins. However, this should not be an issue when analysing airway tissue sections and secretions because Muc5ac and Muc5b are the major polymeric mucins found in equine airway mucus, and tandem mass spectrometry analysis presented here and previously [20] have not identified other polymeric mucins. Furthermore, tandem mass spectrometry analysis of the mucins present in the secretions pooled from the 10 highest mucus score samples did not identify peptides from any polymeric mucin (Muc2, Muc6 nor Muc19) other than Muc5b and Muc5ac (data not shown).

Despite these limitations, our study has highlighted major increases of both Muc5ac and Muc5b mucins. How these changes impact on the mucus properties in terms of barrier function and transport is not known. However, studies performed on human sputum may shed some light on these issues. Several studies have shown that the mucin composition of sputum expectorated from human patients suffering from asthma, chronic obstructive pulmonary disease (COPD) and cystic fibrosis (CF) is altered [24], [25], [26]. Importantly, in a study of COPD, the relative amounts of MUC5AC and MUC5B were shown to differ in smokers with and without airway obstruction [26], suggesting that the mucin composition of mucus is a major factor for efficient mucus transport.

Other studies have analysed the expression of *Muc5ac* in horses with RAO and have yielded contradictory data, with an increase, or no change in *Muc5ac* expression being reported [55], [56]. Increased *Muc5ac* expression seems more likely since the data reporting no change in *Muc5ac* expression were obtained from broncheoalveolar lavage fluid rather than epithelial tissue. Nevertheless, in these two studies, the expression of *Muc5b* was not determined and it is possible that Muc5b alone is responsible for the mucus accumulation in RAO. The work presented here demonstrates that Muc5b is much more abundant than Muc5ac in healthy as well as in diseased horses, which indicates that it is essential to consider both Muc5b and Muc5ac when examining changes in mucus.

To conclude, the work presented here shows that there is an increase in both Muc5ac and Muc5b in the mucus accumulated in equine airways. The critical steps that lead to mucus overproduction remain to be elucidated. Understanding of mechanisms of inflammation and response to pathogens has been greatly advanced by the development of cell culture models and in particular the air-liquid interface model culture of primary airway epithelial cells [57], [58]. Development of this culture model for equine airways epithelial cells, combined with the polyclonal antisera described here would be invaluable for unravelling the biological events leading to mucus overproduction in horses and possibly man.

## Materials and Methods

### Mucin gene expression in salivary glands, trachea and stomach

Equine tissues were dissected within one hour after death from client owned horses euthanased for clinical reasons not relating to the respiratory tract, which demonstrated no clinical or pathological signs of respiratory diseases. Full informed consent was obtained from the owners of the horses following internal ethical review. Additional tissue was obtained post-mortem from an equine abattoir. No horses were euthanased specifically for this study. The tissues were snap frozen in liquid nitrogen, ground into a fine powder using a micro-dismembranator and then transferred to TRI reagent. RNA extraction was then carried out using the phenol/choloroform method of Chomczynski [59] and total RNA was purified using the Rneasy RNA miniprep extraction kit (Qiagen) according to the manufacturer's instructions. The purification included a DNAse I treatment step to remove contaminating DNA. Total RNA concentrations were calculated from absorbance reading at 260 nm using a GeneQuant (Amersham Biosciences) and the RNA quality for 1 representative sample of each tissue was verified using an agilent chip (Agilent). 1 µg of freshly isolated RNA was reverse transcribed using MMLV reverse transcriptase and random hexamers. Briefly, 1 µg of RNA was incubated for 5 minutes at 70°C in the presence of 0.5 µg of random hexamer primers and then cooled on ice. Once cooled, the mix was incubated with a master mix containing 0.5 mM of each dNTP, 24 units of Rnase inhibitor and 200 units of Moloney-murine leukemia virus (M-MLV) reverse transcriptase, for 1 hour at 37°C followed by a denaturation step of 5 minutes at 95°C. All enzymes were from Promega.

Real Time oligonucleotide primers were designed using the primer express 2.0 software (Applied Biosystems) under default parameters and using predicted sequences from the equine genome [20]. The oligonucleotides were: *Muc5b* forward: 5′-GTGGAGCAGAGCAGCGTCTAC-3′, *Muc5b* reverse: 5′-GCTCCAGCAGGGCACTGT-3′, *Muc5ac* forward: 5′-GATTCTGAGCGAGGTCTTCGA-3′, *Muc5ac* reverse: 5′-GGAGCACACCACGTCCAAGT-3′. Oligonucleotides were also designed for two housekeeping genes, succinate dehydrogenase complex subunit A (*SDHA*, sequence: XM_001490889, forward primer: ***5′-*** ACAGAGGAATGGTCTGGAAATACTGA-3′, reverse primer 5′-GTGAGCACCACGTGACTCCCTT-3′), and glyceraldehyde-3-phosphate dehydrogenase (*GAPDH*, sequence XM_001496020, forward primer: ***5′-*** GTGTCCCCACCCCTAACGT-3′, reverse primer: 5′-TCATCGTATTTGGCAGCTTTCTC-3′). The quantitative Real Time PCR assays were performed in triplicate using a TaqMan™ ABI PRISM 7900 (Applied Biosystems). Each 10 µl reaction volume consisting of 5 µl 2X (POWER SYBR master mix (Applied Biosystems), 0.3 µl each of 20 µM forward and reverse primers and 4.6 µl of sample cDNA or water (negative control). Quantitative assays were validated using SYBR Green chemistry. Assays were classed as validated if they had detection ranges of at least five orders of magnitude, with an efficiency of between 90 and 110% as measured by a standard curve slope of between -2.95 and -3.62 (100%  = 3.3). Specificity of primer sets was confirmed by the observation of single amplification products of the expected size and melting temperature in all assays. Expression of each gene was calculated from the geometric mean of the CT of 2 housekeeping genes, GAPDH and SDHA using the 2-ΔCt method [60].

### Polyclonal antisera

Polyclonal antisera were raised in rabbits against peptide sequences located in putative the N-termini of equine Muc5b and Muc5ac (MANeq5b-I; DEDYPTSEKAGGDIEC) and Muc5ac (MANeq5ac-I; GIDSGDFDTLENLR). Two rabbits were immunised per peptide and immunization consisted of four injections of each peptide conjugated to KLH (antisera were commissioned from Eurogentec). Antisera were purified using Protein A. In brief, 25 mg of protein A Sepharose was mixed with 1 ml of antiserum diluted 1∶2 in binding buffer (0.1 M tris-HCl pH 7.5) and mixed overnight at 4°C. The unbound molecules were eluted with PBS and the immunoglobulins were eluted twice using 0.5 ml of 0.1 M glycine solution at pH 5 and 2.5, twice using 0.1 M Tris-HCl pH 8, and finally twice with 100 mM TAE at pH 11. All fractions were collected into tubes containing 50 µl of 1 M Tris-HCl pH 8. The immunoreactivity of each fraction was tested on Western blots of salivary and gastric mucus separated by agarose gel electrophoresis, and by immunohistochemical staining of the relevant tissues.

### Histological and immunohistochemical staining

Epithelial tissues were collected from euthanased horses and immediately fixed in formalin or ethanol. After dehydration with xylene, the samples were then embedded in wax and 5 µm sections cut. Prior to sectioning, tracheal tissue was treated for 1 h with 1∶1 Da Castors solution (5% nitric acid and 50% methanol in water) and Baker's solution (10% glycerol and 50% methanol in water) to decalcify the tissue. Sections from each tissue were stained with periodic acid Schiff's and alcian blue pH 2.5 (PAS-AB). For immunohistochemical staining, sections were dewaxed and microwaved for 5 minutes at 500 W with antigen retrieval solution (10 mM sodium citrate pH 6). The sections were left to cool prior to reduction with 10 mM dithiotreitol (DTT) and alkylation with 25 mM iodoacetamide in 0.1 M Tris-HCl pH 8; each treatment was applied for 30 minutes. Endogenous peroxidase activity was quenched with 3% (v/v) hydrogen peroxide in methanol for 30 minutes at room temperature. The tissue sections were blocked with 10% (v/v) donkey serum and 1% (w/v) bovine serum albumin in PBS (blocking solution) for one hour prior to incubation with protein A-purified mucin-specific antibodies (diluted 1 in 100 in the blocking solution for 1 hour at room temperature). Sections were then incubated for 30 minutes with biotin labelled donkey anti-rabbit IgG (Santa Cruz), diluted 1∶250 in the blocking solution, and then treated with streptavidin (ABC Kit Elite standard, VectaStain) for 30 minutes prior to detection with Diaminobenzidine (Sigma). All sections were counterstained with Harris's haematoxylin and representative pictures were taken using an Axiovision microscope. Goblet cell counts were performed to assess the number of surface epithelial goblet cells that stained with PAS, MANeq5ac-I and MANeq5b-I. ImageJ software was used to assess the percentage of the area of the submucosal glands stained with PAS, MANeq5ac-I and MANeq5b-I. The level of statistical significance was determined using the student's t-test.

### Mucin purification

Mucus samples were collected at an equine abattoir, within 20 minutes after death; these horses were healthy and had no identifiable respiratory disease. The mucus was collected by scraping the stomach and immediately mixed with 8 M guanidinium chloride and the mucins were extracted with gentle stirring for 4 days at 4°C. Horse saliva was collected and treated in an identical fashion. Mucins were purified by caesium chloride density gradient in 4 M guanidinium chloride at a starting density of 1.4 g/ml and centrifuged for 65 h at 40000 rpm in a Beckman Ti45 rotor as previously described [20].

### Anion exchange chromatography

Mucins purified by density gradient centrifugation were reduced and carboxymethylated as previously described [61] using 10 mM DTT (one hour at 37°C) followed by 25 mM iodoacetamide (10 minutes at room temperature in the dark); and dialysed into 6 M urea. The reduced and carboxymethylated mucins were subjected to anion exchange chromatography on a Resource Q column (1 ml × 1 ml, Amersham). Briefly, mucins were bound to the column using 10 mM piperazine pH 5 in 6 M urea. Fractions of 0.5 ml were eluted with an increasing concentration of 0.4 M lithium perchlorate/10 mM piperazine, pH 5 in 6 M urea (0–100%, total volume 30 ml). After chromatography selected fractions were subjected to agarose gel electrophoresis.

### Rate-zonal centrifugation

Mucins purified by density gradient centrifugation were analysed by rate-zonal centrifugation on 6–8 M guanidinium chloride gradients as described previously [20]. Unreduced mucins were centrifuged in a SW40Ti swing-out rotor at 40000 rpm for 2.5 h and reduced mucins at 40000 rpm for 7 h.

### Agarose gel electrophoresis and Western blotting

The samples, dialysed into 6 M urea, were electrophoresed on 0.7% agarose gels in 40 mM Tris acetate, 1 mM EDTA, pH 8.0 containing 0.1% (w/v) SDS. The gels were run for 3 h at 65 V. After electrophoresis, the proteins were vacuum blotted onto nitrocellulose membrane with a vacuum blotter; 40mbar pressure in 0.6 M sodium chloride, 60 mM sodium citrate [62]. Mucins were detected with the equine mucin specific antisera and an alkaline phosphatase conjugated secondary antibody; blots were developed with a NBT/BCIP solution for 1 minute.

### Mass spectrometry for mucin identification

Following trypsin digestion of mucins, peptides were concentrated and desalted using solid phase extraction with ZipTips (Millipore). The peptides were separated using a 0.075×250 mm BEH UPLC column (Waters) on a nanoAcquity liquid chromatography system (Waters) before being automatically analysed on an LTQ Velos ion trap mass spectrometer (Thermo). The data produced was searched using Mascot software (Matrix Science) against a custom database containing SwissProt database (EBI) and equine mucin sequences determined in our laboratory.

### Tracheal wash samples

In order to examine mucin content in the context of mucus hypersecretion, we used aliquots of archived frozen tracheal washes, which had been collected as part of a wider longitudinal study of IAD in National Hunt racehorses conducted by the two authors Drs Cardwell & Newton [36], [37].

For each sample, a mucus score was assigned during endoscopic examination according to Gerber et al [63] (Score 0: no visible mucus, score 1: small amount, isolated flecks, score 2: moderate amounts, multiple larger blobs, and score 3: large amounts visible, larger blobs at least partly confluent). In addition, the types and number of bacteria present in each tracheal wash had been determined immediately after return of samples on ice in a cool box to the laboratory. Briefly, samples were serially diluted in PBS (10^−1^, 10^−2^, 10^−3^ and 10^−4^) and 0.1 ml was spread onto, a selective medium for Streptococci (colistin oxolinic acid blood agar [64]) and anaerobic horse blood agar solid bacteriological media. The total number of colonies of each bacterial species separately and of all bacteria in total were calculated and expressed as log_10_ number of colony forming units per ml (cfu/ml) of undiluted sample.

Drs Cardwell & Newton's study consisted of an overall total of 1184 samples collected monthly from 177 horses during the study [36], [37], with the number of samples from each horse varying between 1 and 15 samples. For the present study, samples with high mucus scores were selected (all those still available); this represented 18 samples collected from 16 different horses (15 samples with a mucus score of 2 and 3 samples with a mucus score of 3). Lower mucus score samples were used as control and corresponded to the other samples collected from the aforementioned 16 horses.

### Relative quantitation of Muc5b and Muc5ac in tracheal wash samples

Each tracheal wash sample was dissolved in 5 volumes of 8 M guanidinium chloride for at least 5 days, and dialysed against 6 M urea. Using a method similar to that published by Kirkham et al [25], several dilutions of each sample, prepared using a Hamilton syringe for accuracy, were analysed by agarose gel electrophoresis followed by Western blotting with the MANeq5b-I and MANeq5ac-I antisera. The antibody response for each dilution was measured using a Biorad Model GS 800 calibrated densitometer.

### Preparation Muc5ac and Muc5b mucin standards

Gastric mucus and saliva were solubilised in 8 M guanidinium chloride and the mucins purified by caesium chloride density gradient purification [20] followed by anion exchange chromatograpy as described above. Refractometry was used to determine to concentration of the mucin solutions, in brief, samples were chromatographed on a Superose 6 column (0.8 cm ×30 cm) eluted at a flow rate of 0.83 ml/min with 0.2 M NaCl/1 mM EDTA. The column effluent was monitored with an in-line Optilab rEX refractometer (Wyatt Technology Corporation, Haverhill, Suffolk, UK) using a dn/dc value of 0.165. Tandem mass spectrometry of trypsin digested mucins (as described above) was used to identify the mucins present in the preparations. The salivary mucin preparation which contained a mixture of Muc5b and Muc19 mucins was further analysed by tandem mass spectrometry to determine the ratio of Muc5b/Muc19. In brief, 3 separate aliquots of the purified mucin solution were digested with trypsin and analysed by tandem mass spectrometry; the number of occasions that each peptide had a spectral match (number of spectra per peptide) was used to determine the most abundant peptides. The mass to charge ratios of each of the three most abundant peptides were used to create extracted ion chromatograms using Xcaliber software (Thermo) and the identity of each peak checked by matching the relevant fragmentation spectra to the database matched versions. The sum peak area or height was calculated for each mucin peptide in each sample and the ratio of these sum values was considered to reflect the relative protein concentrations of the two mucins [38].

### Statistical analyses

SPSS v16 software was used to conduct statistical analyses with P≤0.05 considered statistically significant. The association between the ordinal Muc5ac and Muc5b mucin scores and continuous Muc5b and Muc5ac antibody reactivities was examined by ANOVA. Pearson's correlation test was used to determine the correlation between the reactivities of Muc5ac and Muc5b, as well as the correlation between mucins antibody response and continuous log_10_ bacterial count.

## Supporting Information

Table S1 **Information on samples.** Table of results showing the MANeq5ac-I and the MANeq5b-I antibody reactivities, mucus score and the total bacterial count for each of sample tested.
(PDF)
