# Supplementary material for: Muc5b Is the Major Polymeric Mucin in Mucus from Thoroughbred Horses With and Without Airway Mucus Accumulation
Source: PLoS One. 2011 May 13;6(5):e19678. doi: 10.1371/journal.pone.0019678 (PMC3094342; doi:10.1371/journal.pone.0019678)
Supplement: Table S1 — Information on samples. Table of results showing the MANeq5ac-I and the MANeq5b-I antibody reactivities, mucus score and the total bacterial count for each of sample tested. (PDF) [file pone.0019678.s001.pdf]

Sample number	MANeq5ac-I reactivity	MANeq5b-I reactivity	Mucus score	Total bacterial count
1	0.0000	19.5060	3	5.3384
2	0.0000	0.0000	0	1.6020
3	11.7227	8.3278	2	2.7243
4	0.0531	0.0000	0	0.0000
5	2.1014	0.0000	0	2.6812
6	1.0877	0.0000	0	2.0792
7	44.1465	77.3198	2	3.1847
8	4.5685	16.1960	2	3.1673
9	5.3793	0.0000	0	1.6020
10	0.5000	0.0000	1	2.0792
11	6.5000	1.4541	0	2.9777
12	0.5500	0.0000	1	2.0000
13	1.6668	0.0000	0	2.8977
14	20.0000	38.3181	2	4.0766
15	0.0000	0.0000	0	0.0000
16	34.3167	36.1923	2	2.8062
17	2.9372	0.0000	0	2.1461
18	14.8000	47.0000	2	0.0000
19	8.8000	8.4000	2	3.1004
20	0.0000	2.4120	0	0.0000
21	7.7000	3.0000	2	2.5185
22	0.9792	0.0000	0	0.0000
23	0.0000	0.0000	0	2.7853
24	13.7367	28.0915	2	4.8232
25	58.2402	24.8115	2	2.1761
26	0.0000	0.0000	0	1.9542
27	40.7247	6.2400	3	4.9638
28	0.0000	0.0000	0	0.0000
29	0.0000	0.4000	0	1.0000
30	0.0000	0.4306	0	2.7730
31	73.3286	31.1741	2	5.9783
32	7.9576	15.6000	3	5.7324
33	0.0000	0.0000	0	2.9777
34	0.0000	66.5787	3	7.5441
35	0.0000	0.0000	0	1.0000
36	0.0000	0.0000	0	2.7708
37	0.0000	0.0000	0	2.6434
38	6.8950	0.0000	0	2.6812
39	19.9568	5.8563	0	3.0000
40	11.9718	47.9460	1	3.0334
41	3.5669	0.0000	0	3.6263
42	0.0000	0.0000	0	2.1761
43	4.2799	0.0000	1	3.1931
44	0.0000	0.0000	0	2.6232
45	0.0000	0.0000	1	1.0000
46	0.0000	0.0000	0	1.4771
47	0.0000	0.0000	0	1.0000
48	4.2461	0.0000	1	2.7324

49	0.0000	0.0000	1	2.9085
50	6.7807	46.9447	0	7.5241
51	9.1607	19.5793	0	4.6543
52	0.0000	0.0000	2	4.3358
53	13.5582	83.5566	2	3.0755
54	0.0000	0.0000	1	0.0000
55	5.7265	6.8083	1	2.6628
56	3.1373	0.0000	0	10.0000
57	13.4212	9.7918	1	4.6813
58	0.0000	0.0000	0	1.4771
59	13.4511	10.0554	0	3.4362
60	0.3153	2.5936	2	2.9445
61	0.0000	0.0000	0	0.0000
62	3.4372	0.3295	1	3.2833
63	0.0000	0.0000	1	2.8751
64	46.0991	17.1581	0	4.7561
65	0.0000	-0.0482	0	2.6128
66	0.0000	0.0000	1	4.6304
67	0.0000	0.0000	0	1.7782
68	50.6597	53.2990	1	3.1703
69	11.2406	0.0000	0	2.6232
70	0.0000	0.0000	0	2.1761
71	0.0000	0.0000	0	1.7782
72	0.0000	0.0000	1	0.0000
73	3.1000	0.6232	0	0.0000
74	0.0000	0.0000	0	2.5051
75	0.0000	0.0000	0	3.1959
76	0.0000	0.0000	0	3.1206
77	15.2969	11.1787	0	4.6990
78	1.1613	0.0000	1	2.0792
79	0.0000	0.0000	0	1.7782
80	0.0000	0.0000	0	2.7243
81	0.5904	0.0000	0	2.6902
82	0.0000	0.0000	0	2.2304
83	0.0000	0.0000	0	0.0000
84	0.0000	0.0000	0	1.8451
85	0.0000	1.3374	1	0.0000
86	6.4075	18.5569	2	3.6053
87	14.8036	22.6821	2	2.6812
88	1.5661	1.2239	1	3.1644
89	0.0000	0.0000	0	0.0000
90	0.0000	0.0000	0	1.7782
91	0.0000	0.0000	1	0.0000
92	0.0000	0.0000	0	0.0000
93	69.4575	121.8949	1	4.7172
94	13.0262	32.7260	0	3.4669
95	1.6500	0.0000	0	3.3655
96	0.0000	0.0000	1	2.4314
97	0.0000	0.0000	1	2.5051
98	0.0000	0.0000	1	1.6990

99	0.0000	1.8336	1	1.9542
100	0.0000	0.0000	0	2.1461
101	0.0000	1.3930	0	10.0000
102	0.0000	6.0695	1	1.7782
103	3.7838	27.3000	1	2.4150
104	0.4885	30.0172	1	2.7076
105	2.6408	6.8180	2	2.8513
106	34.2132	27.5671	0	5.0693
107	2.6835	6.9438	0	5.0128
108	13.5681	16.2144	1	4.8148
109	3.0355	5.7985	0	3.5302
110	51.1121	51.7028	0	5.0141
111	0.0000	2.4556	1	1.4771
112	10.8411	12.9503	1	3.3927
113	0.7020	0.0000	1	2.0414
114	59.0739	105.7870	2	3.0719
115	13.1931	43.1708	1	4.3690
116	0.2914	0.6373	1	2.7634
117	0.0000	0.8033	0	1.6990
118	0.0000	0.0000	0	2.0792
119	2.9602	0.2325	0	2.1461
120	7.7685	16.9410	0	2.4624
121	3.7601	4.1977	1	1.6990
122	5.0150	4.4912	1	4.7278
123	0.0000	0.0000	0	0.0000
124	0.0000	0.0000	0	0.0000
125	16.1120	33.0431	2	0.0000
126	0.9857	3.1177	1	2.8325
127	0.0000	0.4316	1	1.9542
128	0.0000	0.0000	0	0.0000
129	0.0000	0.0000	1	0.0000
130	0.0000	0.0000	0	1.9031
131	0.0000	0.1094	0	1.0000
132	0.0000	0.0000	0	3.0374
133	0.0000	0.0000	0	3.0043
134	29.8781	16.1285	1	4.9494
135	22.9877	9.3399	0	4.7860
136	20.7835	22.2621	1	2.3802
137	19.9250	14.1923	1	3.0212
138	108.4258	59.8320	2	2.9685
139	47.5913	60.8828	1	3.0294
140	0.0000	0.0000	1	1.6990
141	0.0000	0.0000	0	1.7782
142	0.0000	0.0000	1	1.6020
143	46.9294	79.7457	1	1.9031
144	4.5529	7.6837	0	1.3010
